# Evolution of research in hernia repair materials: A 20-year bibliometric and visualization study

**DOI:** 10.1097/MD.0000000000043021

**Published:** 2025-06-27

**Authors:** Miaofeng Wang, Wei Chen, ZhuHong Xie

**Affiliations:** a Department of General Surgery, Shaoxing Central Hospital (The Central Affiliated Hospital, Shaoxing University), Shaoxing, Zhejiang Province, China; b Department of General Surgery, The First Affiliated Hospital of Anhui Medical University, Hefei, Anhui Province, China.

**Keywords:** bibliometric analysis, biological meshes, hernia repair, nanotechnology, synthetic meshes, tissue engineering

## Abstract

**Background:**

Hernia repair is a prevalent surgical procedure globally, with increasing demand due to factors such as aging populations, obesity, and previous surgeries. The evolution of hernia repair materials and techniques has significantly impacted surgical outcomes, with advancements focusing on reducing recurrence rates and complications. This study aims to provide a comprehensive bibliometric analysis of research trends in hernia repair materials from 2004 to 2024.

**Methods:**

Data were collected from the Web of Science database to quantitatively assess publication trends, influential authors, journals, and collaborative networks. Bibliometric tools such as VOSviewer and CiteSpace were used to analyze keyword co-occurrence, research hotspots, and the evolution of study themes. The analysis focused on the progression from synthetic to biological and composite meshes, alongside the development of minimally invasive techniques.

**Results:**

The study revealed a steady increase in research output, peaking in 2020, with the United States leading in innovation. Initial research focused on synthetic meshes such as polypropylene; however, recent trends include the exploration of biological, composite, and absorbable materials. Advances in laparoscopic and robotic-assisted surgeries were also prominent, reflecting a shift toward patient-centered approaches. Emerging technologies such as nanotechnology and 3D-printed meshes were identified as promising future directions.

**Conclusion:**

This bibliometric analysis demonstrates the evolution and diversification of hernia repair materials and techniques, emphasizing the need for continued innovation. Future research should focus on optimizing patient outcomes through advanced technologies such as tissue engineering and nanotechnology, which have the potential to overcome existing limitations and enhance clinical efficacy.

## 1. Introduction

Hernia repair is a fundamental aspect of abdominal wall surgery, representing one of the most common procedures performed worldwide.^[1,2]^ As the global population ages and risk factors such as obesity, chronic cough, and previous surgeries increase in prevalence, the demand for effective hernia repair continues to grow. Over the past several decades, techniques for hernia repair have evolved significantly, largely driven by advancements in the materials used for reinforcement.

Historically, hernia repairs relied on sutured closures, which were often associated with high recurrence rates and significant patient discomfort.^[3,4]^ The introduction of synthetic meshes revolutionized hernia surgery, allowing for tension-free repair techniques that greatly reduced recurrence rates and improved outcomes.^[5]^

Synthetic meshes, such as those made from polypropylene or polyester, provided the necessary mechanical strength for effective hernia closure but were not without complications, including chronic pain, infection, and adhesion formation. More recently, biological meshes have emerged as an alternative, particularly in cases where synthetic meshes may pose a higher risk of complications, such as contaminated surgical fields or patients with poor tissue quality.^[6,7]^ Despite their promise, biological meshes also present challenges, such as higher costs and varying rates of integration with host tissues.

The evolution of hernia repair materials has significantly impacted surgical outcomes and patient quality of life, particularly in minimizing complications and recurrence rates. To optimize these outcomes and enhance patient care, it is essential to understand how these materials and techniques have developed over time. This study conducts a comprehensive bibliometric analysis of research on hernia repair materials, leveraging major scientific databases such as Web of Science to quantitatively assess publication trends, key journals, influential authors, and collaborative networks in the field. Using bibliometric tools such as VOSviewer and CiteSpace, the study maps out research hotspots, tracks emerging trends, and highlights influential research groups, providing insights into how knowledge in hernia surgery has evolved. The findings aim to identify cutting-edge materials and techniques while outlining future research directions that could fill current gaps and guide clinical practice. By systematically analyzing the development and dissemination of hernia repair innovations, this study not only offers a historical perspective but also serves as a strategic guide for advancing standards in hernia repair, ultimately contributing to improved patient outcomes.

## 2. Methods

### 2.1. Data sources

Data for this analysis were gathered from the Web of Science databases. The search included all articles related to hernia repair materials, published between 2004 and 2024. Keywords such as “hernia,” “mesh,” “hernia repair,“ “prosthetic materials,” and “laparoscopic hernia surgery” were used to ensure comprehensive coverage of relevant studies.

### 2.2. Search strategy and selection criteria

The search terms included combinations of “Hernia” OR “Inguinal Hernia” OR “Ventral Hernia” OR “Incisional Hernia” OR “Umbilical Hernia” OR “Epigastric Hernia” OR “Femoral Hernia” OR “Abdominal Wall Hernia” OR “Hiatal Hernia” AND “Repair” OR “Hernia Repair” OR “Hernioplasty” OR “Herniorrhaphy” AND “Mesh” OR “Surgical Mesh” OR “Prosthesis” OR “Prosthetic Materials” OR “Synthetic Mesh” OR “Biological Mesh” OR “Absorbable Mesh” OR “Non-absorbable Mesh” OR “Composite Mesh.” Only peer-reviewed original research articles and review papers were included, while conference abstracts and nonpeer-reviewed papers were excluded to maintain data quality.

### 2.3. Analytical tools

Bibliometric analyses were performed using VOSviewer and CiteSpace. These tools were utilized to create visual representations of citation relationships, co-authorship networks, and keyword co-occurrences, and to identify clusters of research activity. Citation indicators, including total citations and H-index, were also analyzed to determine the influence of individual publications and researchers in the field.

## 3. Results

### 3.1. Publication trends over time and active countries

**Figure 1.**
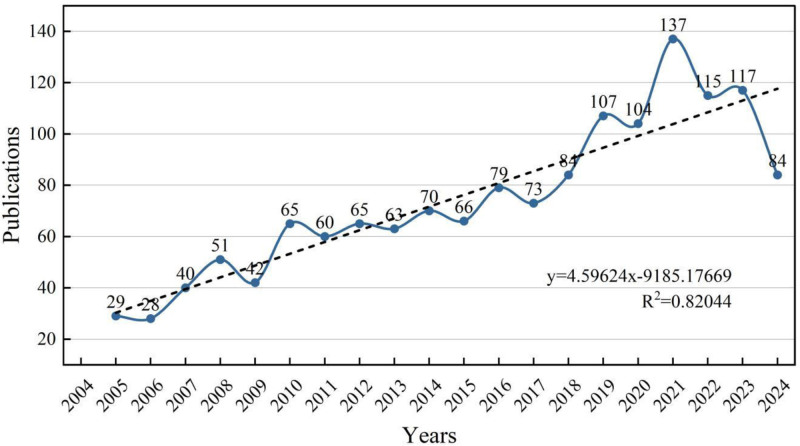
Distribution of articles published on hernia repair materials by year.

Figure 1 presents the trend in the number of publications on hernia repair materials from 2004 to 2024. Initially, there was gradual growth from 2004 (29 publications) to 2011 (65 publications). Following fluctuations from 2012 to 2016, a significant upward trend occurred, peaking in 2020 (137 publications).

Despite subsequent decreases, publication counts in recent years (2021–2024) remained higher than earlier periods, indicating sustained interest in this research area. The linear trend line (*R*^2^ = 0.82044) indicates a positive overall growth trajectory, suggesting expanding academic attention and ongoing advancements in hernia repair materials over the past 2 decades.

**Figure 2.**
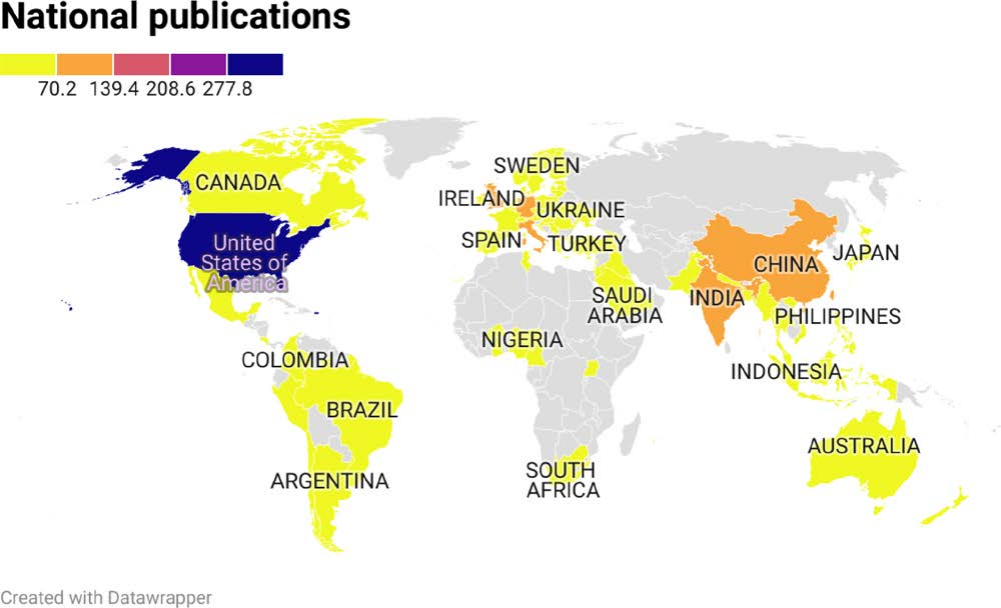
Global productivity maps illustrating the distribution of published articles on hernia repair materials across different countries.

Figure 2 illustrates the distribution of articles by country. The top 20 countries contributing the most publications are led by the United States, which accounts for 347 articles, representing 23.5% of the total. This is followed by Taiwan with 131 articles (8.8%) and China with 131 articles (8.8%).

**Figure 3.**
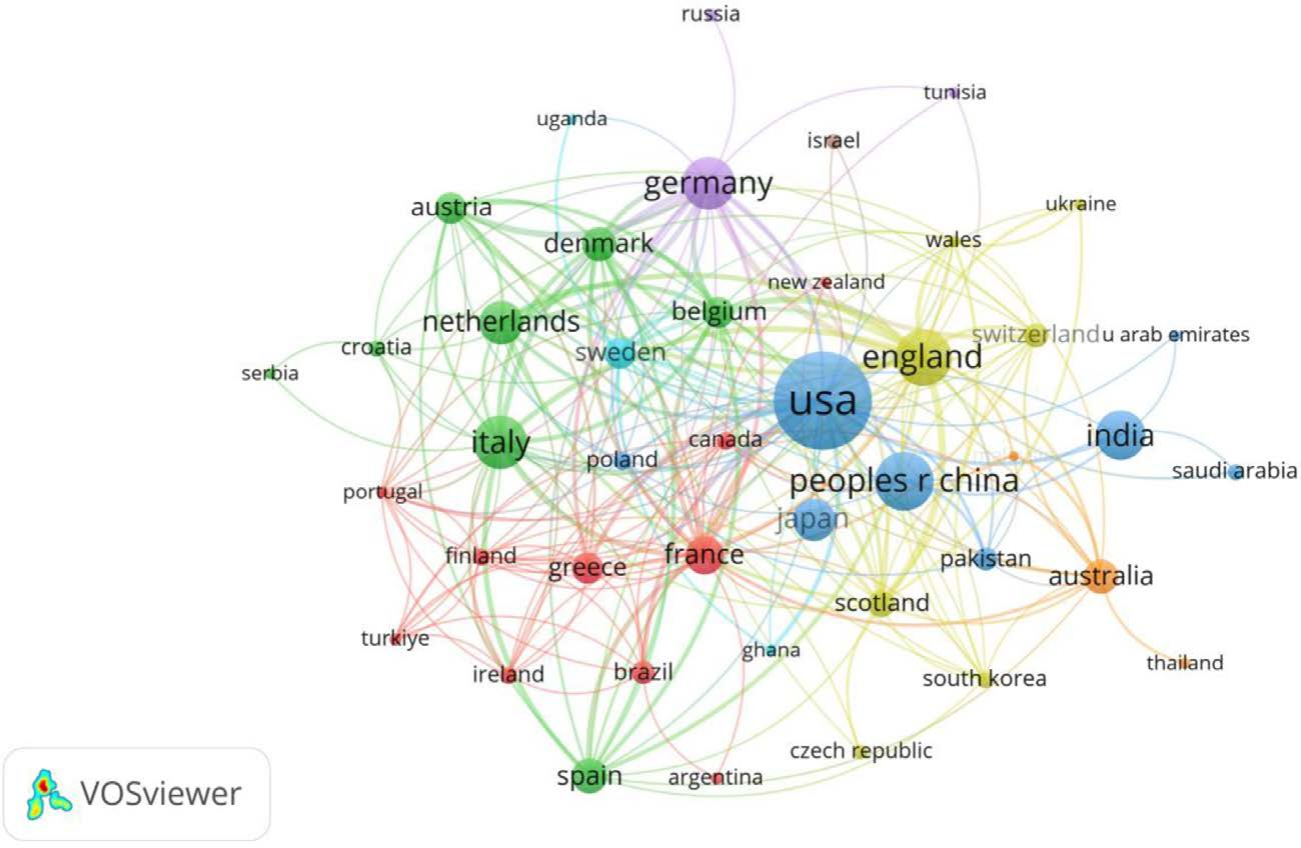
visualizes international collaboration networks in hernia repair materials research, highlighting major contributing countries such as the USA, China, and England, and illustrating their cooperative relationships through bibliometric clusters.

Figure 3 is a bibliometric network visualization created using VOSviewer, illustrating international collaboration patterns in research on hernia repair materials. The nodes represent countries, with the size of each node indicating the volume of publications from that country, and the links between them depicting co-authorship networks. Larger nodes such as the USA, China, and England suggest these countries have the most significant output and influence in the field. The thickness of the links represents the strength of collaboration, with thicker lines indicating stronger cooperative relationships. The different colors cluster countries into collaborative groups or regions, highlighting the predominant partnerships and research networks across geographical and scientific communities. Notable clusters include the USA’s strong connections with European countries such as England, Germany, and France, and China’s prominent interactions with Asian and other international collaborators. This visualization effectively demonstrates the global nature of research in hernia repair materials, with substantial intercontinental and regional collaborations driving the scientific output.

**Figure 4.**
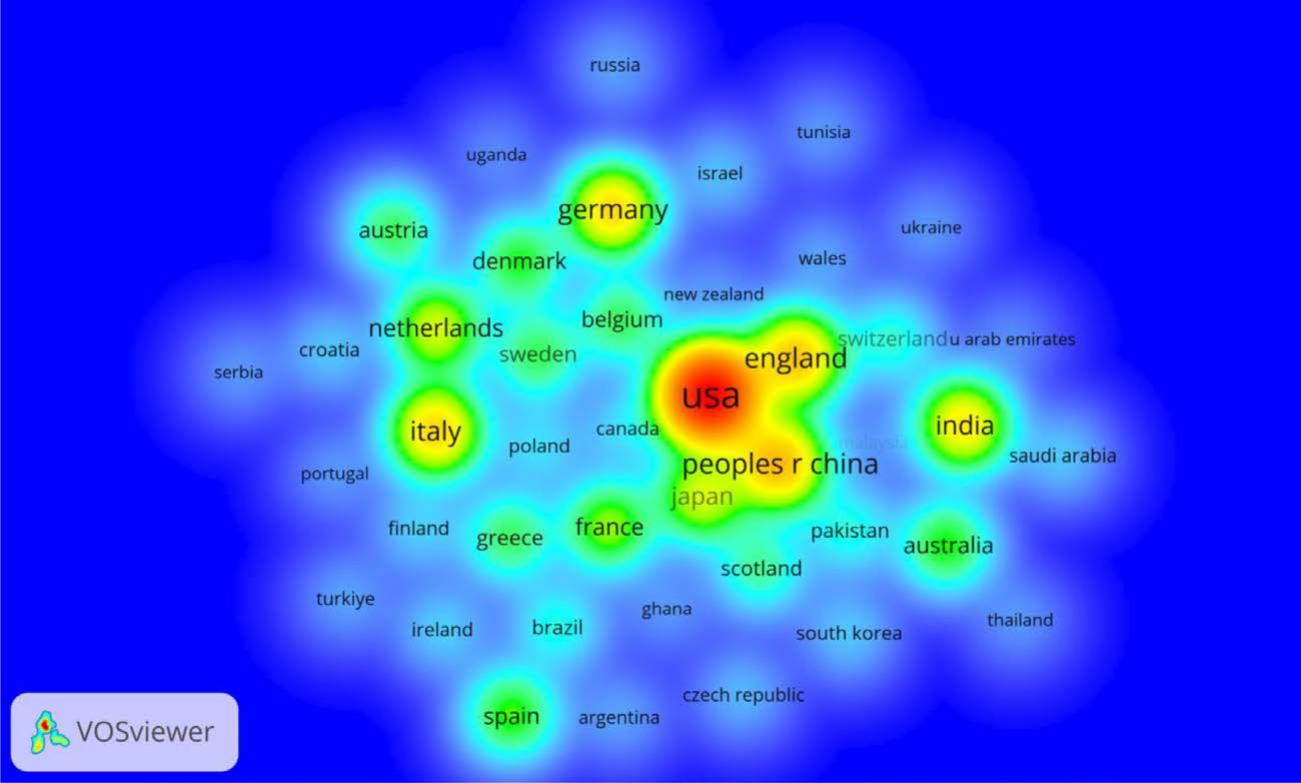
The heat map illustrates the global research intensity and collaboration in hernia repair materials, with the USA, China, and major European countries emerging as the primary contributors and collaborative hubs.

Figure 4 is a heat map visualization generated by VOSviewer, representing the intensity of research activity and collaboration among countries in the field of hernia repair materials. The color gradient, ranging from blue (low activity) to red (high activity), highlights the most influential countries. The USA is marked in red, indicating it as the leading contributor and collaborator in this domain. Other countries such as China, England, Germany, and Italy show significant activity, as represented by yellow and green shades, reflecting their prominent roles and collaborations. The heat distribution emphasizes the central hubs of research and collaboration networks, showcasing the dominance of North America, Europe, and Asia in driving advancements in hernia repair materials. This visualization effectively illustrates global research intensity and cooperation, providing insights into key regions leading scientific output and development.

### 3.2. Leading journals and influential publications

**Figure 5.**
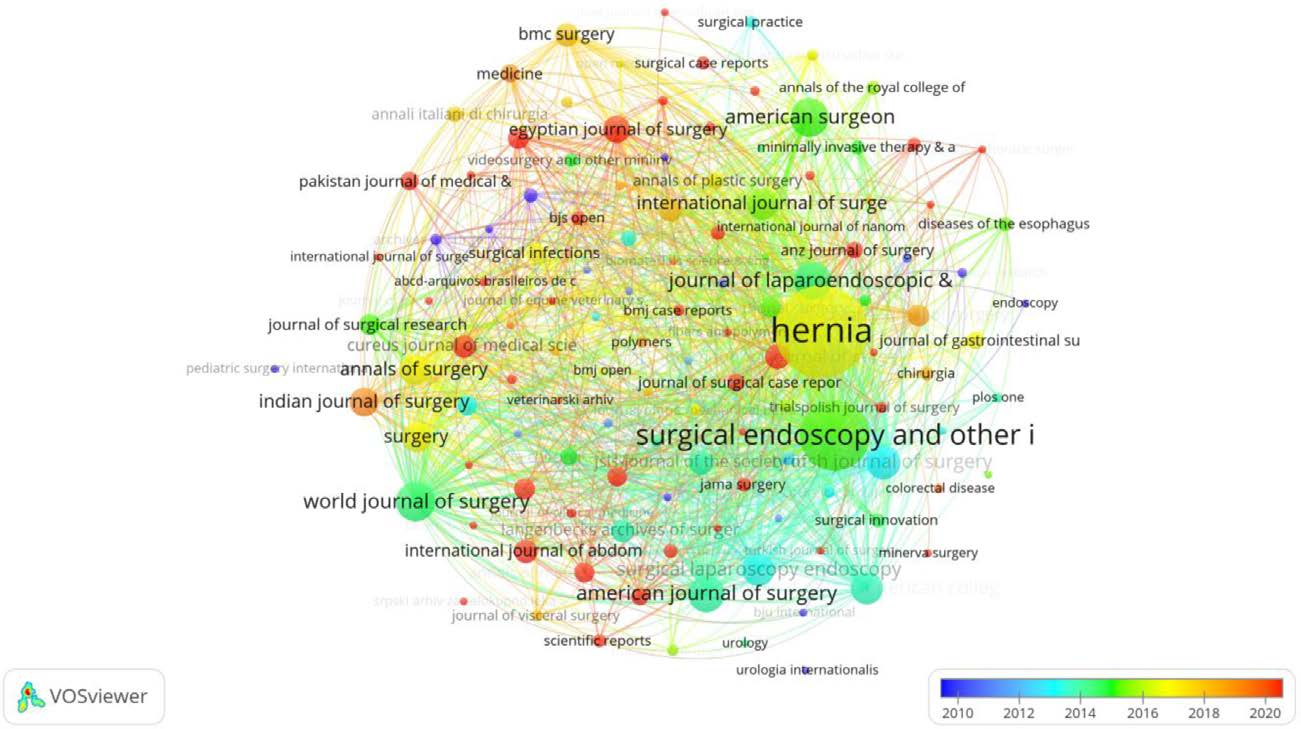
The visualization illustrates the co-citation network and journal prominence in hernia repair materials research, highlighting key journals and the evolution of research trends over time based on publication year.

Figure 5 is a bibliometric network visualization created using VOSviewer, showing the co-citation relationships and journal prominence in the field of hernia repair materials research. The nodes represent journals, with the size indicating the number of citations received, and the connections between them reflecting co-citation links. The larger nodes, such as “Hernia,” “Surgical Endoscopy and Other Interventional Techniques,“ and “Journal of Laparoendoscopic & Advanced Surgical Techniques,” indicate these journals as key sources of influential publications within this field. The color gradient, ranging from blue to red, reflects the average publication year, with blue representing older publications (2010) and red representing more recent ones (2020). This gradient reveals the evolution and focus of research over time, with newer research being increasingly concentrated in journals specialized in surgery and laparoscopic techniques. The visualization emphasizes the centrality and importance of specific surgical journals and highlights the dynamic and evolving nature of hernia repair materials research, illustrating how co-citation patterns align with emerging trends and influential publications in the field.

Table 1 ranks the top 10 journals in hernia repair research by publications. Hernia leads with 242 articles, 4072 citations, and the highest link strength (58,676). Other key journals include Surgical Endoscopy and other interventional techniques (138 articles) and World Journal of Surgery (44 articles), highlighting their influence in the field. Table 2 lists the top 10 most cited studies in hernia repair, summarizing their objectives, authors, publication details, and citation metrics. The highest-cited article, published in Annals of Surgery (2006), has 370 citations. Key themes include mesh durability, postoperative outcomes, and technique comparisons, underscoring their significant influence in advancing hernia repair methodologies.

**Table 1:** Top 10 journals by number of publications.

Journal name	Number of articles	Number of citations	Total link strength
Hernia	242	4072	58,676
Surgical Endoscopy and Other Interventional Techniques	138	4058	39,872
World Journal of Surgery	44	1404	15,510
American Surgeon	42	348	7826
Journal of Laparoendoscopic & Advanced Surgical Techniques	41	363	9987
American Journal of Surgery	39	1588	14,975
British Journal of Surgery	33	2066	14,410
Journal of the American College of Surgeons	30	1150	11,108
Surgical Laparoscopy Endoscopy & Percutaneous Techniques	30	335	9251
International Journal of Surgery	28	658	11,251

**Table 2:** The 10 most cited articles.

Title	Objective of the study	Authors	Year of publication	Journal	Total Number of citations	Mean citation per year
Biologic prosthesis reduces recurrence after laparoscopic paraesophageal hernia repair: a multicenter, prospective, randomized trial	This trial was designed to study the value of a biological prosthesis, small intestinal submucosa (SIS), in Laparoscopic paraesophageal hernia repair.	Brant K. Oelschlager	October 2006	Annals of Surgery	370	19.47
Biologic Prosthesis to Prevent Recurrence after Laparoscopic Paraesophageal Hernia Repair: Long-term Follow-up from a Multicenter, Prospective, Randomized Trial	To determine the long-term durability of biologic mesh-buttressed repair.	Brant K. Oelschlager	October 2011	Journal of the American College of Surgeons	310	22.14
The lightweight and large porous mesh concept for hernia repair	To compare two mesh designs in hernia surgery, arguing that the lightweight large porous mesh is superior in reducing long-term complications and improving postoperative quality of life.	Bernd Klosterhalfen	January 2005	Expert Review of Medical Devices	304	15.2
Comparison of endoscopic procedures vs Lichtenstein and other open mesh techniques for inguinal hernia repair – A meta-analysis of randomized controlled trials	To compare endoscopic and open mesh techniques for inguinal hernia repair, using meta-analysis to evaluate their advantages and disadvantages regarding complications, recovery, and recurrence rates.	Schmedt, C. G.	February 2005	Surgical Endoscopy and other Interventional Techniques	292	14.6
Chronic pain after mesh repair of inguinal hernia: a systematic review	The objectives are to review the incidence, severity, and consequences of chronic pain and its etiologies.	Simon Nienhuijs	September 2007	American Journal of Surgery	290	16.11
Which mesh for hernia repair?	To guide surgeons in selecting the appropriate mesh by outlining these properties and discussing their impact on biocompatibility, infection risk, and patient outcomes.	Brown, C.N.	May 2010	Annals of the Royal College of Surgeons of England	269	17.93
Randomized clinical trial assessing impact of a lightweight or heavyweight mesh on chronic pain after inguinal hernia repair	To compare pain of any severity at 12 months after inguinal hernia repair with a partially absorbable lightweight mesh (LW group) or with a non-absorbable heavyweight mesh (HW group).	O’Dwyer, P. J.	February 2005	British Journal of Surgery	248	12.4
Mesh location in open ventral hernia repair: A systematic review and network meta-analysis	To identify the optimal mesh placement location for open ventral hernia repair (OVHR) by using systematic review and meta-analysis, concluding that the sublay position is most effective in minimizing hernia recurrence and surgical site infections.	Holihan, J. L.	January 2016	World Journal of Surgery	239	26.56
Meta-analysis of randomized controlled trials comparing open and laparoscopic ventral and incisional hernia repair with mesh	To compare open and laparoscopic ventral and incisional hernia repair with mesh	Forbes, S. S.	August 2009	British Journal of Surgery	236	14.75
Mesh-related infections after hernia repair surgery	Treatment of mesh infection.	Falagas, M. E.	January 2005	Clinical Microbiology and Infection	211	10.55

The results are based on the search carried out for articles until October 2024.

**Figure 6.**
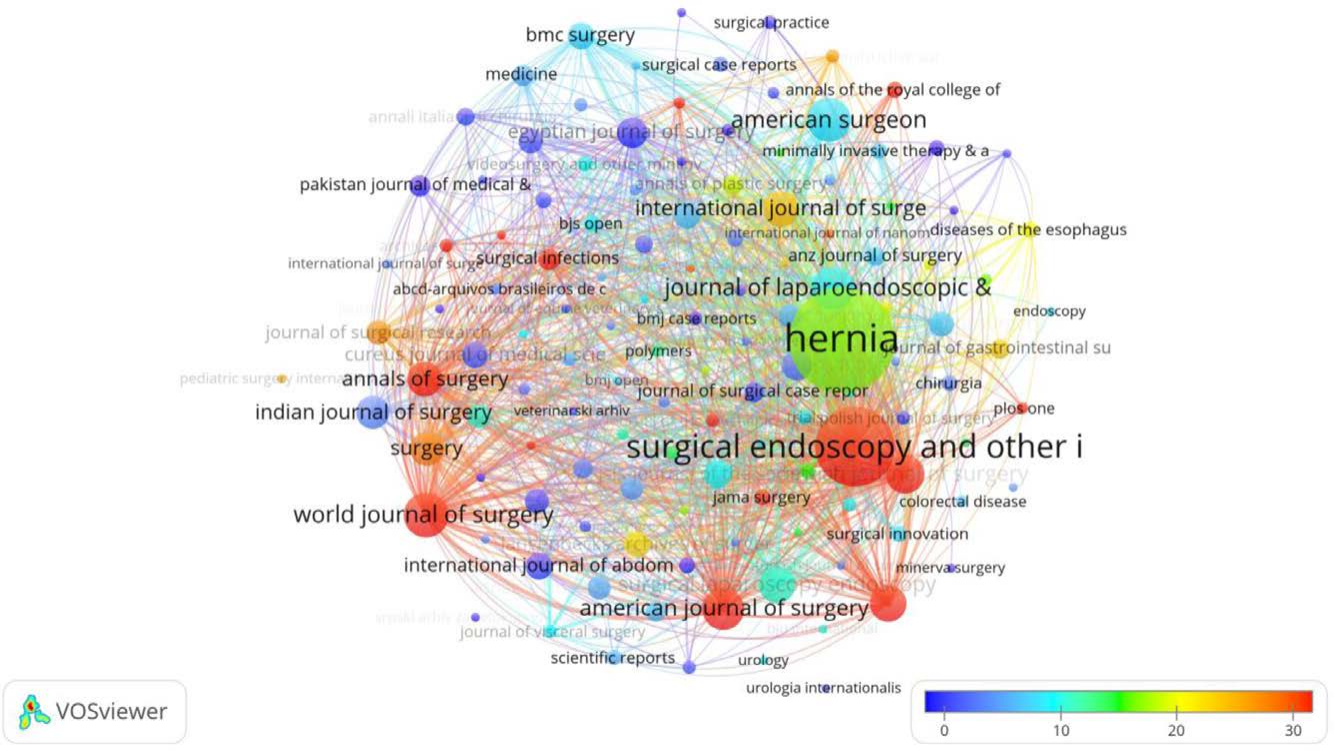
A co-citation network of journals in hernia repair materials research, highlighting key influential journals and their interconnectedness based on citation frequency and impact.

Figure 6 is a co-citation network visualization generated using VOSviewer, focusing on journals related to hernia repair materials research. Each node represents a journal, with the node size corresponding to the frequency of citations it receives. The network’s intricate web of connections shows co-citation relationships, indicating how often journals are cited together, thereby revealing their relevance and influence within the field. Prominent journals such as Hernia, Surgical Endoscopy and Other Interventional Techniques, and Journal of Laparoendoscopic & Advanced Surgical Techniques are larger and centrally positioned, highlighting their significance in disseminating key research findings. The color coding ranges from blue to red, reflecting the average number of citations, where red indicates highly cited journals, emphasizing their impact. The visualization effectively captures the core journals and their interconnectivity, demonstrating how knowledge in hernia repair materials research is shared, expanded, and built upon in the scholarly community. This comprehensive network highlights the multidisciplinary and collaborative nature of the field, showcasing leading sources that contribute to its advancement.

### 3.3. Keyword analysis

**Figure 7.**
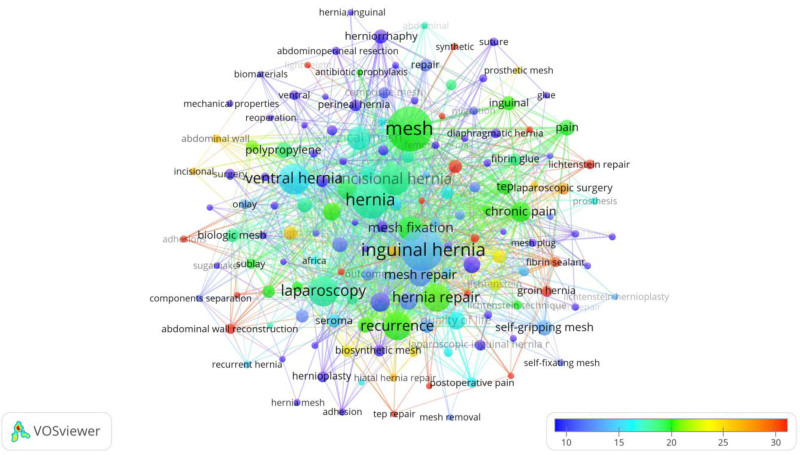
A keyword-based network visualization map, identifying the most frequently cited topics in hernia repair materials.

Figure 7 is a keyword co-occurrence network visualization created using VOSviewer, focusing on the most frequently associated terms in hernia repair materials research. Each node represents a keyword, with its size indicating the frequency of occurrence within the literature, and the links between nodes illustrating co-occurrence relationships, highlighting how often these terms appear together in studies.

Prominent terms such as “mesh,” “inguinal hernia,” “hernia repair,” “laparoscopy,” and “recurrence” are larger and centrally positioned, reflecting their central importance and frequent discussion in the field. The color gradient from blue to red indicates the average number of co-occurrences, with warmer colors (yellow to red) representing higher co-occurrence counts, showing the evolution of key research focuses. This network effectively maps the primary themes and areas of emphasis, including different types of hernias, surgical techniques, and mesh types (e.g., “biologic mesh,” “synthetic mesh”). The visualization reveals the interconnected nature of research topics, emphasizing the complexity and multidimensionality of hernia repair studies and the emphasis on improving surgical outcomes and techniques.

Table 3 highlights the top 20 keywords with the strongest citation bursts in hernia repair research. “Mesh,” “Inguinal hernia,” and “Hernia” are the most frequent, indicating focus areas. Keywords such as “Laparoscopy,” “Chronic pain,” and “Quality of life” reflect evolving themes emphasizing surgical techniques and patient outcomes.

**Table 3:** Top 20 keywords with strong citation bursts.

Keyword	Occurrences	Total link strength
Mesh	236	686
Inguinal hernia	207	539
Hernia	162	427
Incisional hernia	114	280
Ventral hernia	114	317
Laparoscopy	107	305
Recurrence	95	316
Hernia repair	91	189
Mesh fixation	72	189
Surgical mesh	66	167
Mesh repair	59	138
Chronic pain	49	165
Laparoscopic	44	145
Ventral hernia repair	41	87
Inguinal hernia repair	38	94
Hiatal hernia	37	93
Quality of life	37	108
Polypropylene mesh	33	69
Polypropylene	29	78

### 3.4. Emerging trends and research hotspots

**Figure 8.**
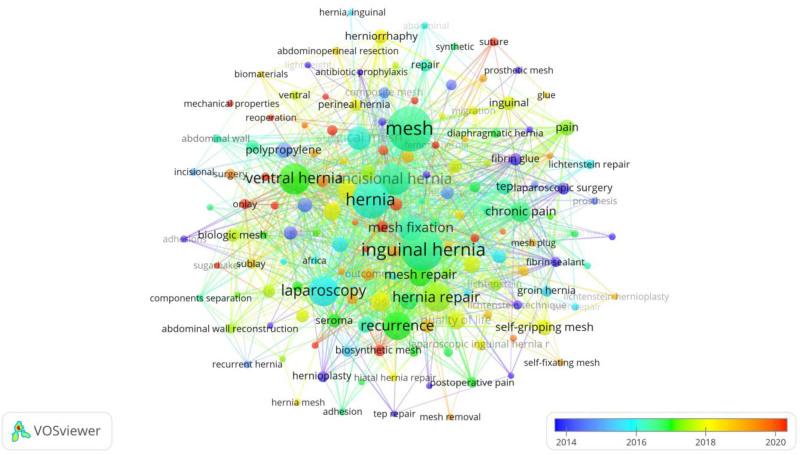
A network visualization map based on keyword analysis, highlighting the evolution of past and current trends in hernia repair materials.

The keyword co-occurrence network illustrates that research on hernia repair materials centers around several key aspects: material selection and biocompatibility, comparison of different surgical techniques, and prevention and management of postoperative complications. With advances in minimally invasive surgery and biologic materials, hernia repair is increasingly focusing on reducing postoperative pain and improving patient quality of life. The focus on reducing recurrence also remains central, with efforts aimed at discovering more effective repair methods. Future research is likely to continue exploring new materials, such as biosynthetic meshes, and optimizing personalized surgical approaches. The trend network visualization map performed to identify trend topics is shown in Figure 8.

## 4. Discussion

This bibliometric study comprehensively evaluates research trends in hernia repair materials from 2004 to 2024, revealing significant developments in the field. The findings demonstrate a steady increase in publications, peaking in 2020, and reflect the global demand for innovation in hernia surgery to improve patient outcomes and surgical techniques. The discussion below addresses the key trends, advances in materials and techniques, emerging research directions, and limitations of this study.

### 4.1. Temporal trends and factors influencing research output

The temporal analysis shows a consistent increase in the number of publications on hernia repair materials, reaching a peak in 2020. This surge is likely correlated with technological advancements in medical devices, refined surgical techniques, and heightened awareness of postoperative complications such as chronic pain and recurrence. Although a slight decline in publication volume has been observed in recent years, the overall output remains significantly higher than in the early stages, indicating that hernia repair remains an active and evolving field of research.

The dominance of the United States as the leading contributor highlights its pivotal role in driving surgical innovations and developing new materials for hernia repair. This is also evidenced by the journal Hernia being a central platform for knowledge dissemination, further reinforcing its influence in advancing research and clinical practice in this domain.

### 4.2. Evolution of research focus and keyword co-occurrence analysis

The keyword co-occurrence analysis reveals a dynamic evolution in research themes over the past 2 decades. Initially, research was primarily focused on synthetic mesh materials, such as polypropylene, which became the gold standard for hernia repair due to its strength and durability. However, as clinical practice evolved, there was a noticeable shift toward exploring the broader implications of mesh use, including its biocompatibility and long-term impact on patient quality of life.

In recent years, research has expanded to include topics such as laparoscopic and robotic-assisted surgeries, chronic pain management, and recurrence rates.^[8]^ This shift reflects a growing emphasis on comprehensive treatment strategies that not only focus on the mechanical aspects of hernia repair but also consider patient-centered outcomes. This evolution highlights the increasing complexity of hernia surgery and the need for holistic approaches that balance efficacy, safety, and long-term patient satisfaction.

### 4.3. Advances in hernia repair materials and surgical techniques

#### 4.3.1. Synthetic and biological meshes

Synthetic meshes, particularly those made from polypropylene, have been widely adopted and remain a cornerstone of modern hernia repair due to their proven efficacy in reducing recurrence rates.^[9]^ Despite their benefits, complications such as chronic pain, mesh-related infections, and adhesion formation remain critical issues, limiting their widespread acceptance in some patient populations. In response to these challenges, biological meshes were developed, offering a biocompatible alternative for patients with contaminated surgical fields or poor tissue quality. These meshes, derived from human or animal tissue, aim to integrate better with host tissue and reduce foreign body reactions.

However, the use of biological meshes is not without limitations. High costs, variability in integration rates, and inconsistent long-term outcomes are major barriers to their broader application.^[10]^ The development of composite meshes, which combine the properties of synthetic and biological components, and absorbable meshes designed to degrade over time, represent significant steps toward optimizing the balance between durability and biocompatibility. These innovations highlight the ongoing pursuit of the ideal hernia repair material, one that minimizes complications while maximizing patient benefits.

#### 4.3.2. Evolution in surgical techniques

The evolution of surgical techniques has paralleled advancements in hernia repair materials. The shift from traditional open surgeries to minimally invasive approaches, such as laparoscopic and robotic-assisted procedures, represents a milestone in reducing patient recovery time and postoperative complications.

Laparoscopic techniques such as transabdominal preperitoneal and totally extraperitoneal repairs have become widely adopted for various hernia types, offering reduced pain and quicker recovery.^[11,12]^ More recently, robotic-assisted surgery has shown promise, particularly for complex or recurrent hernias, by enhancing precision and reducing the risk of chronic pain through improved visualization and dexterity.

### 4.4. Emerging trends and future research directions

Future research is expected to continue focusing on reducing postoperative pain and recurrence rates while improving the quality of life for patients.^[13]^ Innovations such as nanotechnology and tissue engineering hold the potential to revolutionize hernia repair materials.^[14]^ For instance, nanotechnology could be used to develop antimicrobial mesh coatings, which have shown promise in preliminary studies for reducing infection rates.^[15]^ Additionally, advances in tissue engineering and 3D printing technology may allow for the creation of customized, patient-specific meshes tailored to individual anatomical needs, thereby minimizing recurrence and enhancing clinical outcomes.^[16]^ Evaluations of novel materials, such as biosynthetic and fully absorbable meshes, will also be crucial to address the long-term complications associated with existing synthetic and biological options.^[17]^

### 4.5. Limitations of the study

While this study offers valuable insights into global trends in hernia repair materials, several limitations exist. A key limitation of this study is the lack of patient-centered outcomes, such as quality of life and recurrence rates, due to data constraints. The exclusive use of the Web of Science database may have omitted relevant studies from other databases such as PubMed or Embase, potentially compromising the analysis’s comprehensiveness. The focus on English- language, peer-reviewed articles introduces language bias, excluding significant non-English research. Moreover, bibliometric indicators such as citation counts, while reflective of research impact, do not necessarily correlate with clinical relevance or evidence quality. Future research integrating bibliometric data with patient-centered outcomes, including recurrence rates and quality of life, would provide a more holistic evaluation of hernia repair materials’ effectiveness.

## 5. Conclusion

In summary, this bibliometric analysis elucidates the evolution of hernia repair materials and techniques, demonstrating the field’s progression toward more refined and patient-centric approaches. The study highlights the need for continued innovation in materials and surgical strategies, emphasizing the importance of individualized treatment plans that optimize safety, efficacy, and patient satisfaction. Moving forward, integrating advanced technologies such as nanotechnology and tissue engineering could be instrumental in overcoming existing challenges and improving clinical outcomes in hernia repair.

## Author contributions

**Conceptualization:** Miaofeng wang, Wei Chen.

**Data curation:** Miaofeng wang, Wei Chen.

**Formal analysis:** Miaofeng wang, Wei Chen.

**Investigation:** Zhuhong Xie.

**Writing—review & editing:** Zhuhong Xie.
